# Semi-Correlations for the Simulation of Dermal Toxicity

**DOI:** 10.3390/toxics13040235

**Published:** 2025-03-23

**Authors:** Andrey A. Toropov, Alla P. Toropova, Alessandra Roncaglioni, Emilio Benfenati

**Affiliations:** Istituto di Ricerche Farmacologiche Mario Negri IRCCS, Via Mario Negri 2, 20156 Milan, Italy; andrey.toropov@marionegri.it (A.A.T.); alessandra.roncaglioni@marionegri.it (A.R.); emilio.benfenati@marionegri.it (E.B.)

**Keywords:** acute dermal toxicity, SAR, SMILES, Monte Carlo method, semi-correlation, CORAL software

## Abstract

The skin is the primary pathway for harmful substances to enter the body and a susceptible target organ, making compound-induced acute dermal toxicity a significant health risk. In this work, the possibility of modelling dermal toxicity using so-called semi-correlations is studied. Semi-correlations are a specific case of correlations, where one variable takes only two values. For example, 0 denotes the absence of activity (e.g., dermal toxicity), and 1 denotes the presence of activity. The described computational experiments can be carried out by interested readers using the freely available software CORAL.

## 1. Introduction

The skin is the primary pathway for harmful substances to enter the body and a susceptible target organ, making compound-induced acute dermal toxicity a significant health risk [1]. The research on acute dermal toxicity has consistently been a crucial component in assessing the potential risks of human exposure to active ingredients in cosmetics in particular, pharmaceuticals mainly for topical use, most substances of common use for consumers, and all substances for occupational exposure. However, it is difficult to directly identify the acute dermal toxicity of potential compounds through animal experiments alone [2].

The growing popularity of studies on various skin dermal toxicity is explained by the increase of medicinal agents assessed as safe and effective due to their natural origin and long history of use, which are, however, dangerous sometimes. The inherent natural quality of these agents does not guarantee their safety, as evidenced by the risks associated with their use [3]. It is important to test and confirm the safety and efficacy of these agents for use. According to the Organisation for Economic Co-operation and Development (OECD) guidelines, acute toxicity refers to adverse reactions observed shortly after application to the skin of a single dose of the test substance [4]. Dermal toxicity may cover several endpoints, such as irritation and sensitization, but also related effects due to systemic exposure. The era of big data and high-throughput screening technology has led to the generation and collection of vast amounts of experimental dermal toxicity data in publicly available databases. These data have stimulated the development and validation of computational simulations that are further used to predict dermal toxicity in a variety of fields and organisms [5,6,7,8].

One can expect new, more complex technologies and treatments that will require an expansion of the list of substances of interest, generally speaking, for all endpoints, and in particular for acute dermal toxicity [9]. This process can progressively result in updated versions of the database, but the new data may be introduced or not, and the frequency of the updated versions varies [10]. In all cases, the number of substances we are exposed to is much higher and increasing more rapidly than the updating procedure of the databases. Thus, there is a need to adopt further approaches to cope with dermal toxicity, different from experimental assays, while waiting for the experimental results. In silico models may be a valuable solution.

There are two options for constructing in silico models, depending on the presentation of the corresponding data. The regression model, where a continuous range of values from a certain minimum to a certain maximum is studied (simulated). Another option is the prediction of two states: the substance is active or inactive [11].

**Figure 1:**
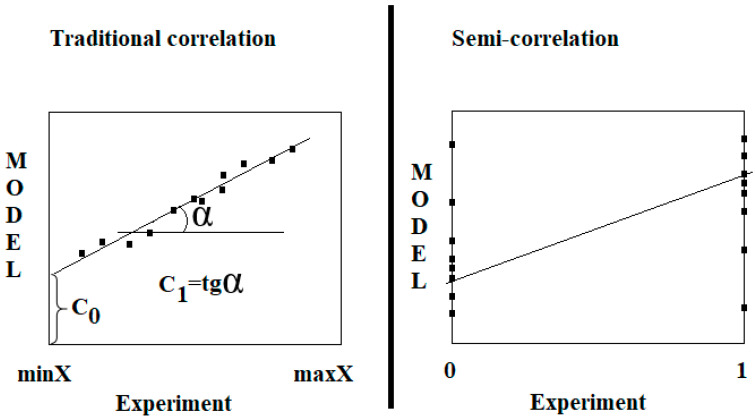
Comparison of the traditional correlation and the semi-correlation.

For the case of linear regression models, the least squares method is often used, which allows, based on the series of experimental and calculated values of the endpoint being studied, to find the regression coefficients C_0_ and C_1_, the meaning of which is presented in Figure 1. For the case of classification models, the least squares method is not appropriate. However, as it turned out, this method can be applied in this case if we accept the possibility of using so-called semi-correlations.

The idea of semi-correlation is an attempt to use algorithms designed to handle “ordinary” regressions for the case when the simulated quantity takes two values, interpreted as activity (which is denoted, for example, by 1) and lack of activity (which is denoted, for example, by 0). Here, the semi-correlations are used to develop a categorical model of acute dermal toxicity. The CORAL-2024 software (http://www.insilico.eu/coral, Accessed on 21 March 2025) is a tool for building semi-correlations. Simplified molecular input-line entry systems (SMILES) [12] are used to represent the molecular structure and calculate the corresponding structural descriptors [13,14].

## 2. Materials and Methods

### 2.1. Data

Data on acute dermal toxicity of 2616 compounds have been taken from the literature [15]. The effect summarises results related to different endpoints, both topical (skin sensitization, skin irritation and corrosion, and eye irritation and corrosion) and systemic (acute oral toxicity, acute inhalation toxicity, and acute dermal toxicity). The total number of active compounds (acute dermal toxicity observed) is 382. Thus, there is an imbalance in the data. For constructing binary classification models in general, and for constructing such models using the semi-correlation method, it is preferable to use balanced data where the numbers of active and inactive connections are at least approximately balanced (coincident). Considering this, the random lists containing 382 active compounds (in all splits, these are the same compounds) and 382 inactive compounds were selected randomly ten times. Every time, non-identical lists of inactive compounds have been used. For the splits, a similar approach has been used.

The approach under consideration includes the following steps. First, the available data (thus, 382 active and 382 inactive substances) are divided into four sets. The sets are designated as active training (≈25%), passive training (≈25%), calibration (≈25%), and validation (≈25%) sets. The latter remains invisible until the end of model development. This allows the set to be used for the final assessment of the predictive potential of the resulting model. The active training set is used as a basis for model development by calculating the so-called correlation weights for the molecular features involved in model building. As the outcome of the active training set, there are the selected correlation weights. During these calculations, compounds assigned to the passive set act as permanent checkers, checking whether the model is good for compounds outside the active training set. The calibration set is designed to catch the moment of overfitting when the improvement in statistical quality on the training sets is accompanied by a deterioration in statistics on the calibration set. The considered split into the specified subsets is carried out by means of the Las Vegas algorithm, aimed at obtaining the division most favourable for the calibration set, with the hope that such a division will also be favourable for the external validation set. The selection of the best split for the calibration set is repeated in ten random attempts, assessing the differences between the splits. While the active and passive training sets are initial steps in the model’s development, only with the results from the calibration set all parameters are optimised. Thus, the results from the calibration set are those relevant to describe the statistical values of the model, and not the results of the active and passive training sets, which are shown below. Moreover, the use of the calibration set is very useful to avoid overfitting, focussing attention on the results from substances not yet used in the model’s development.

However, the statistical evaluation of the calibration set provides information on the robustness of the model, but not on its predictivity when new substances are evaluated. For this purpose, the validation set is used. Thus, the validation set provides the check considering substances that have not been used in the model development. Obviously, different partitions lead to different statistical results, and the average values should be considered as the quality indicator.

### 2.2. Model

The model of dermal toxicity is defined as

(1) $$MODEL=C_{0}+C_{1}\times DCW(T,N)$$

(2) $$DCW\left(T,N\right)=\sum CW(S_{k})+\sum CW({SS}_{k})+\sum CW({SSS}_{k})$$

The descriptor DCW(T,N) is calculated with correlation weights (CW) of SMILES attributes. Here, three types of SMILES attributes are considered: Sk is the SMILES-atom, i.e., one symbol (‘C’, ‘N’, ‘S’, etc.) or a group of symbols (‘Cl’, ‘%11, ‘@@’, etc.), which lose meaning if the symbols are separated; SSk is a pair of SMILES-atoms which are neighbours in SMILES notation; and SSSk is a sequence of three SMILES-atoms which are neighbours in the SMILES notation. T and N are parameters of the Monte Carlo optimization: T is the threshold to define rare SMILES attributes, which should be removed from the simulation process and active (non-rare) SMILES attributes, which should be involved in the simulation process; N is the number of epochs of the process of the Monte Carlo optimization aimed at defining correlation weights applied in the calculation with Equation (2).

**Figure 2:**
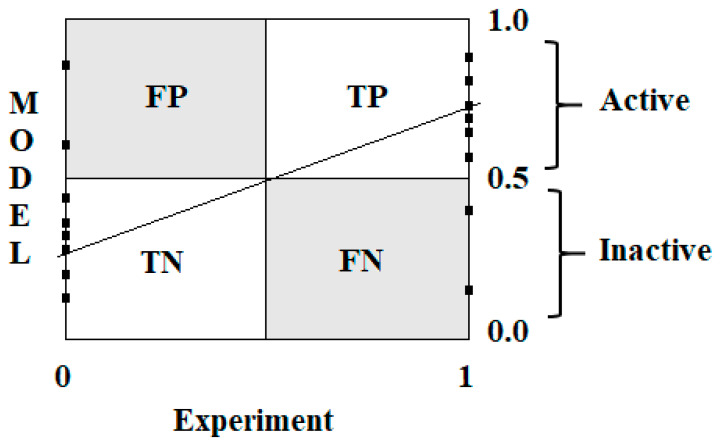
Graphical representation of applying semi-correlation in the classification model.

However, when using such a model, it is not a numerical value that is needed, but a categorical definition in the format of “active or inactive” [13]. The formation of these two categories is numerically expressed by Equation (3). Graphically, these categories are presented in Figure 2. The threshold 0.5 is selected as the middle value between inactive (i.e., 0) and active (i.e., 1). Indeed, if we imagine that activity ranges from 0 to 1, we may have all possible values between these two cases, and this situation is addressed by regression models. Instead, in our case, to address a categorical output, we simply define that all substances with values below 0.5 are inactive, and the others are active. This is the easiest solution, which has been applied in other cases, for instance, by the US EPA predictive platform T.E.S.T. (https://www.epa.gov/comptox-tools/toxicity-estimation-software-tool-test, Accessed on 21 March 2025), to define whether a predicted substance is mutagenic or not. Thus, this value should not be associated with a specific toxicological potency, but it is simply used to introduce a mathematical parameter functional in our semi-correlation algorithm.

(3) $$CATEGORY\left(SMILES\right)=\left\{\begin{array}{c} \;\;1\;\;\left(active\right),if\;MODEL\geq 0.5\\ 0\left(inactive\right),if\;MODEL<0.5 \end{array}\right.$$

The effectiveness of binary classification was assessed according to the following statistical characteristics:

(4) $$Sensitivity\;(Sens)=\frac{TP}{TP+FN}$$

(5) $$Specificity\;(Spec)=\frac{TN}{TN+FP}$$

(6) $$Accuracy\;(Acc)=\frac{TP+TN}{TP+FP+FN+TN}$$

(7) $$Mathew\;correlation\;coefficient\;(MCC)=\frac{TP\times TN-FP\times FN}{\sqrt{\left(TP+FP)(TP+FN)(TN+FP)(TN+FN\right)}}$$

### 2.3. Monte Carlo Optimization

The Monte Carlo method was used to calculate the correlation weights. Two objective functions are considered in this study: TF_0_ and TF_1_.

(8) $${TF}_{0}=r_{AT}+r_{PT}-\left|r_{AT}-r_{PT}\right|\times 0.1$$

(9) $${TF}_{1}={TF}_{0}+{IIC}_{C}\times 0.3$$

$r_{AT}$ and $r_{PT}$ are determination coefficients between the experimental and calculated endpoint values for the active and passive training sets, respectively.

IIC_C_ is the index of ideality of correlation calculated with data on the calibration set as follows [14]:

(10) IICC=rCmin⁡(MAEC−,MAEC+)max⁡(MAEC−MAEC+)

(11) $$\min\left(x,y\right)=\left\{\begin{array}{c} x,\;if\;x<y\\ y,otherwise \end{array}\right.$$

(12) $$\max\left(x,y\right)=\left\{\begin{array}{c} x,\;if\;x>y\\ y,otherwise \end{array}\right.$$

(13) MAEC−=1N−∑∆k,N is the number of ∆k−<0

(14) MAEC+=1N+∑∆k,N is the number of ∆k+≥0

(15) $$\Delta_{k}={observed}_{k}-{calculated}_{k}$$

r_c_ is the correlation coefficient between the observed and calculated values of the endpoint on the calibration set. Observed and calculated are the corresponding values of ‘y’ applied to define the corresponding categories (active/inactive).

The use of the index of ideality of correlation has a rather strong effect on the Monte Carlo process in the case of constructing conventional regression models. The essence of this effect is improving the statistical quality of correlations on the calibration set, even though it may be accompanied by some decrease in statistical quality on the training sets (usually on both: active and passive). As we clarified above, the model in the stage of the active and passive training sets is not mature, and only when all parameters are optimized is the model built up. This occurs using the calibration set. The use of the index of ideality of correlation, which is still within the process of model building, privileges the role of the calibration set versus the results of the initial steps of model building. The calibration set has a broader assessment of all the factors, which may affect the model, compared to the active and passive sets, which only use the initial correlation weights applied to part of the chemicals. As we explained, relying on the active and passive sets introduces the risk of overfitting, since apparently good results on these sets may not be replicated using new substances. Conversely, the index of ideality of correlation forces the system towards the best results obtained on the calibration set.

### 2.4. Applicability Domain

The applicability domain, calculated with Equation (3), defines the so-called statistical defects of SMILES attributes. These defects can be calculated as follows:

(16) $$d_{k}=\frac{\left|{P(A}_{k})-{P\prime (A}_{k})\right|}{N\left(A_{k}\right)+N\prime (A_{k})}+\frac{\left|{P(A}_{k})-{P\prime \prime (A}_{k})\right|}{N\left(A_{k}\right)+N\prime \prime (A_{k})}+\frac{\left|{P\prime (A}_{k})-{P\prime \prime (A}_{k})\right|}{N\prime \left(A_{k}\right)+N\prime \prime (A_{k})}$$

where P(A_k_), P′(A_k_), P″(A_k_) are the probabilities of *A_k_* in the active training, passive training, and calibration sets, respectively; N(A_k_), N′(A_k_), and N″(A_k_) are frequencies of A_k_ in the active training, passive training, and calibration sets, respectively. The statistical SMILES-defects (D_j_) are calculated as follows:

(17) $$D_{j}=\sum_{k=1}^{NA}d_{k}$$

where NA is the number of non-blocked SMILES attributes in the SMILES.

A SMILES falls in the applicability domain if

(18) $$Dj<2*\bar{D}$$

The $\bar{D}$ is the average value of the Dj on the active training set.

### 2.5. Mechanistic Interpretation

Having the numerical data on the correlation weights of codes applied in quasi-SMILES, which were observed in several runs of the Monte Carlo optimization, one can extract three categories of these codes:

(i) Codes that have a positive value for the correlation weight in all runs. These are promoters of endpoint increase;
(ii) Codes that have a negative value for the correlation weight in all runs. These are promoters of endpoint decrease;
(iii) Codes that have both negative and positive values for the correlation weight in different runs of the optimization. These are codes with unclear roles (one cannot classify these features as promoters of increase or decrease for the endpoint).

## 3. Results

**Figure 3:**
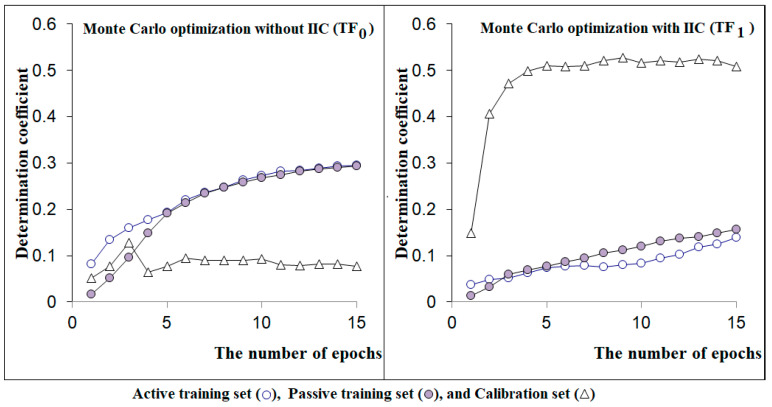
The comparison of histories of the Monte Carlo optimizations for the case of target functions TF_0_ and TF_1_.

The statistical results obtained from the described computational experiments are reported (i) for the case of applying the target function TF_0_ in Table 1; and (ii) for the case of applying target function TF_1_ in Table 2; as well as in Figure 3.

Figure 3 shows that the sequence of optimizations using TF_0_ and TF_1_ is significantly different; for the case of optimization without the correlation ideality index (TF_0_), the threshold is T = 3; the number of training epochs N=3 was adopted, i.e., descriptor DCW (3, 3) was used. For the optimization with the target function TF_1_, descriptor DCW (3, 15) was used. One can see from Figure 3 that for semi-correlations obtained with the target function TF_1_, there is a distribution of statistical quality in favour of the calibration set.

Table 1 presents the statistical characteristics of the models obtained using Monte Carlo optimization with the target function TF_0_. The Mathew correlation coefficient (MCC) is the most informative measure of the predictive potential of classification models. An MCC value above 0.5 indicates that the model is good, while a value close to 0 means that the model is poor. It can be seen that the MCC values for the active training set and passive training set are generally significantly higher than the MCC values for the calibration and validation sets. The values change depending on the split, and there are low values in some cases, indicating problems.

Table 2 presents the statistical characteristics of the models obtained using Monte Carlo optimization with the target function TF_1_. It can be seen that when using TF_1_ as the objective function, the situation changes: For the training samples, the MCC values are quite low, but at the same time, the MCC values for the calibration set and the validation set are quite high. The reason for this is that the simulation process, due to the use of the correlation ideality index, turns towards increasing the statistical quality of the calibration set, without forcing the improvement of the statistics on the training sets. When the process of optimisation occurs on the active and passive training sets, overtraining occurs, and thus the model is not able to extract rules of general value. The final development of the model occurs during the calibration set step. Thus, the statistics for the calibration set are those to be used to characterise the results of the model internally, while the results on the validation set represent the situation expected when the model is used externally. Fortunately, as can be seen from Table 2, the increase in MCC for the calibration set is accompanied by an increase in MCC for the validation set, which confirms that the model is mature and valid, and thus can be used for predictions.

The models obtained using TF_1_ are as follows:

MODEL = 0.3787 + 0.04072 × DCW(3, 15)(19)

MODEL = 0.3512 + 0.04326 × DCW(3, 15)(20)

MODEL = 0.3868 + 0.03540 × DCW(3, 15)(21)

MODEL = 0.5030 + 0.05521 × DCW(3, 15)(22)

MODEL = 0.5911 + 0.07688 × DCW(3, 15)(23)

MODEL = 0.5841 + 0.04142 × DCW(3, 15)(24)

MODEL = 0.5362 + 0.02138 × DCW(3, 15)(25)

MODEL = 0.5474 + 0.04579 × DCW(3, 15)(26)

MODEL = 0.5758 + 0.05128 × DCW(3, 15)(27)

MODEL = 0.5357 + 0.02854 × DCW(3, 15)(28)

The check performed for the applicability domain has shown that the applicability domain observed for external validation sets according to the statistical defect values, on average, is more than 80%.

Table 3 presents the results of five probes of the Monte Carlo optimization using target function TF_1_. Table 3 presents the observed promoters of increase and decrease in the probability of dermal toxicity in the five optimization probes along with their prevalence in the active and passive training sets and the calibration set, depending on the sign of the correlation weight, i.e., positive or negative. Dermal toxicity is a complex phenomenon. Therefore, to discern truly reliable bases for the mechanistic interpretation of models, it is necessary to process data on the behaviour of larger molecular fragments than those collected in Table 3. Nevertheless, it should be noted that there are fragments for which a stable role in impacting the probability of dermal toxicity is observed. No less important, among these, there are fragments characterized by significant prevalence in the training and the calibration sets.

## 4. Discussion

The approach under consideration requires a large amount of data (100 or more substances). It is impossible to use the described methodology for 10 or 15 substances. In addition, a balance between the numbers of active and inactive substances is necessary. As Box said [16], “all models are wrong, but some are useful”. Can the approach under consideration be assessed as useful?

The reliability and reproducibility of the statistical quality of the forecast obtained by using semi-correlations are confirmed across ten significantly different distributions of available data in the training samples and the validation set. It should be noted that the filling of inactive molecules for each of the mentioned separations was carried out from a total array containing 2234 substances. In other words, the probability of random success of the described approach should be considered a very unlikely event.

The use of the correlation ideality index deserves special attention. The fundamental feature of the stochastic process implemented using the above-mentioned index is its focus on the forecast description for the calibration set. As noted above, this is done regardless of the statistical values for the training samples (active and passive). The success of this technology is due to the fact that the above-mentioned sets are nevertheless taken into account. However, in the case of Monte Carlo optimization with TF_1_, the role of the training sets in generating a model through the correlation weights is exploited and optimised, giving a strong role to the results on the calibration set. It is assumed that good statistical quality on the calibration set should be accompanied by good statistical quality for the external set, which uses substances unknown in the model development process.

The traditional accepted concept of QSPR/QSAR analysis requires the same or at least similar statistical quality of models for all samples used (training and validation). In particular, models should not be overfitted, which is a problem [17]. In the case of simulating endpoints using the described stochastic processes, at the first stage of optimization, the main components (affecting the correlation weights) are molecular fragments and molecules exhibiting average behaviour. Using these data, the optimization algorithm arrives at a model consistent between the training and calibration samples. However, there is a risk of overly optimizing the weights for molecular fragments exhibiting “non-standard” and “non-average” behaviour observed in the training sets. Their optimization leads to an improvement in statistical quality only for the training set. At the same time, the statistical quality of the model for the calibration set decreases [17]. To address this issue, the application of the correlation ideality index blocks the growing influence of non-standard and atypical components in the optimization process. In some cases, this may result in a situation that is apparently a paradoxical model, where the statistical quality for training samples is significantly lower than that for the calibration set, and the validation set exists. This is a semantic aspect because what we refer to as training sets in our modelling approach are only the initial sets, representing the preliminary phases of the modelling. Instead, the results from the calibration set are close to the results for the training set in other models, which do not split the initial set into several subsets. This situation has been shown and discussed in several works where the CORAL-2024 software (https://www.insilico.eu/coral, Accessed on 21 March 2025) is used [18,19,20,21,22,23,24,25].

The analysis of Table 3 shows that the increase in the probability of acute dermal toxicity is caused by the branching of the carbon skeleton, as well as the presence of chlorine, oxygen and nitrogen atoms, and double bonds. A decrease in the probability of acute dermal toxicity is caused by the presence of nitrogen atoms with a triple bond, as well as sulphur atoms with a double bond. The complexity of the mechanisms of dermal toxicity has been discussed many times [26,27,28,29,30,31,32]. Some agreement with the assumptions about the mechanisms of dermal toxicity and the structural alerts presented in Table 3 is observed. The presence of chlorine atoms can also implicate dermal toxicity [28], and oxygen (and other) atoms may indicate the possibility of oxidative stress, also in relation to the branching of the carbon skeleton and polyaromatic rings [29,30,31,32].

The approach discussed has been used to model other endpoints, but this is the first time that the semi-correlation method has been used to model dermal toxicity.

The approach considered is convenient for the practical construction of the models in that its implementation requires only SMILES and experimental data without involving additional descriptors. For interpreting the results, this approach provides a convenient opportunity to assess the relevance of various molecular features according to their distribution in training and the validation sets. Finally, it provides an opportunity for a convenient statistical version of the mechanistic interpretation of the results obtained.

A comparison of the models obtained here with the models proposed in the literature is presented in Table 4. The statistical quality of these models is quite comparable.

We note that the results in Ref. [15] are particularly interesting because in that study authors used exactly the same substances that we used, since we derived the set of substances from that paper. This offers the opportunity to compare the results obtained using the random forest algorithm, as in the original work, with those from our new algorithm. Random forest is a complex machine learning approach, which requires building many parallel models, which are then used collectively. Our algorithm is much simpler and easier, relying on a single model. Another important difference is that the CORAL software does not require the calculation of molecular descriptors. In the original paper, several descriptors were used jointly: two-dimensional Morgan fingerprints, MACCS keys, and the Mordred descriptor. CORAL simply needs the SMILES, and the characters used in the SMILES are used to construct the model, as described above. The results shown in Table 4 for the model as in Ref. [15] were obtained using five-fold external cross-validation. These values may be compared with the results that we obtained using ten splits on the external validation set. The average sensitivity that we obtained is 0.88, which is higher than the 0.74 obtained in Ref. [15]. The average specificity that we obtained is 0.87, which is higher than the 0.78 obtained in Ref. [15]. Thus, in the specific case of the use of exactly the same substances, we obtained better results.

## 5. Conclusions

Semi-correlations may provide a basis for developing qualification models for acute dermal toxicity. The reliability of the suggested approach has been checked with ten random splits. This proves the consistency and robustness of our approach. The applicability domain and mechanistic interpretation of these models have been suggested and discussed. The resulting model showed good statistical performance for acute dermal toxicity based on information about the substance using its molecular representation as SMILES. One can repeat the computational experiments described using freeware and instructions available on the Internet (http://www.insilico.eu/coral, Accessed on 21 March 2025).

## Figures and Tables

**Table 1:** The statistical characteristics of models observed for ten splits in the case of the target function TF_0_.

Split	Set *	Sens	Spec	Acc	MCC	TN	TP	FP	FN	All
1	A	0.6526	0.6875	0.6702	0.3404	62	66	30	33	191
	P	0.6744	0.7944	0.7409	0.4730	58	85	22	28	193
	C	0.6630	0.8061	0.7368	0.4749	61	79	19	31	190
	V	0.6239	0.7407	0.6737	0.3613	68	60	21	41	190
2	A	0.8229	0.7604	0.7917	0.5845	79	73	23	17	192
	P	0.7755	0.7957	0.7853	0.5710	76	74	19	22	191
	C	0.7727	0.7864	0.7801	0.5583	68	81	22	20	191
	V	0.7900	0.7333	0.7632	0.5245	79	66	24	21	190
3	A	0.7579	0.8144	0.7865	0.5734	72	79	18	23	192
	P	0.7364	0.7317	0.7344	0.4643	81	60	22	29	192
	C	0.7340	0.6875	0.7105	0.4219	69	66	30	25	190
	V	0.7711	0.8224	0.8000	0.5935	64	88	19	19	190
4	A	0.7708	0.8105	0.7906	0.5817	74	77	18	22	191
	P	0.6346	0.8023	0.7105	0.4385	66	69	17	38	190
	C	0.6907	0.6915	0.6911	0.3822	67	65	29	30	191
	V	0.6941	0.7103	0.7031	0.4025	59	76	31	26	192
5	A	0.8125	0.7660	0.7895	0.5792	78	72	22	18	190
	P	0.7157	0.8778	0.7917	0.5970	73	79	11	29	192
	C	0.5275	0.7374	0.6368	0.2713	48	73	26	43	190
	V	0.6452	0.8889	0.7708	0.5529	60	88	11	33	192
6	A	0.8000	0.7684	0.7842	0.5687	76	73	22	19	190
	P	0.7976	0.7593	0.7760	0.5528	67	82	26	17	192
	C	0.7667	0.7200	0.7421	0.4861	69	72	28	21	190
	V	0.8053	0.6835	0.7552	0.4919	91	54	25	22	192
7	A	0.7604	0.7708	0.7656	0.5313	73	74	22	23	192
	P	0.8023	0.6442	0.7158	0.4476	69	67	37	17	190
	C	0.6374	0.4400	0.5340	0.0788	58	44	56	33	191
	V	0.6055	0.5854	0.5969	0.1892	66	48	34	43	191
8	A	0.7474	0.7732	0.7604	0.5208	71	75	22	24	192
	P	0.7473	0.7778	0.7632	0.5253	68	77	22	23	190
	C	0.5729	0.7684	0.6702	0.3479	55	73	22	41	191
	V	0.6800	0.7582	0.7173	0.4385	68	69	22	32	191
9	A	0.7128	0.7188	0.7158	0.4315	67	69	27	27	190
	P	0.7723	0.7419	0.7577	0.5145	78	69	24	23	194
	C	0.5612	0.5217	0.5421	0.0830	55	48	44	43	190
	V	0.5281	0.6238	0.5789	0.1524	47	63	38	42	190
10	A	0.8105	0.8387	0.8245	0.6493	77	78	15	18	188
	P	0.8481	0.8716	0.8617	0.7173	67	95	14	12	188
	C	0.6800	0.7553	0.7165	0.4359	68	71	23	32	194
	V	0.7130	0.7558	0.7320	0.4658	77	65	21	31	194

* A = active training set; P = passive training set; C = calibration set; V = validation set; TN = true negative; TP = true positive; FN = false negative; FP = false positive; All = the total number of compounds in a set.

**Table 2:** The statistical characteristics of models observed for ten splits in the case of optimization with target function TF_1_.

Split	Set *	Sens	Spec	Acc	MCC	TN	TP	FP	FN	All
1	A	0.6421	0.6458	0.6440	0.2879	61	62	34	34	191
	P	0.6279	0.6542	0.6425	0.2809	54	70	37	32	193
	C	0.9239	0.8776	0.9000	0.8012	85	86	12	7	190
	V	0.8349	0.8765	0.8526	0.7050	91	71	10	18	190
2	A	0.6875	0.6875	0.6875	0.3750	66	66	30	30	192
	P	0.7245	0.7634	0.7435	0.4879	71	71	22	27	191
	C	0.9205	0.8738	0.8953	0.7919	81	90	13	7	191
	V	0.9000	0.8000	0.8526	0.7057	90	72	18	10	190
3	A	0.6211	0.7320	0.6771	0.3553	59	71	26	36	192
	P	0.6273	0.7073	0.6615	0.3312	69	58	24	41	192
	C	0.9149	0.8646	0.8895	0.7801	86	83	13	8	190
	V	0.9277	0.8411	0.8789	0.7627	77	90	17	6	190
4	A	0.6771	0.7263	0.7016	0.4038	65	69	26	31	191
	P	0.5769	0.7907	0.6737	0.3720	60	68	18	44	190
	C	0.9072	0.8511	0.8796	0.7599	88	80	14	9	191
	V	0.8706	0.9065	0.8906	0.7781	74	97	10	11	192
5	A	0.7292	0.7447	0.7368	0.4738	70	70	24	26	190
	P	0.6569	0.7778	0.7135	0.4357	67	70	20	35	192
	C	0.7692	0.7677	0.7684	0.5366	70	76	23	21	190
	V	0.8495	0.8889	0.8698	0.7394	79	88	11	14	192
6	A	0.7053	0.7368	0.7211	0.4423	67	70	25	28	190
	P	0.6905	0.7222	0.7083	0.4109	58	78	30	26	192
	C	0.8889	0.8900	0.8895	0.7785	80	89	11	10	190
	V	0.9115	0.8734	0.8958	0.7849	103	69	10	10	192
7	A	0.6354	0.6354	0.6354	0.2708	61	61	35	35	192
	P	0.6395	0.6731	0.6579	0.3118	55	70	34	31	190
	C	0.9011	0.8900	0.8953	0.7905	82	89	11	9	191
	V	0.8624	0.9024	0.8796	0.7589	94	74	8	15	191
8	A	0.6000	0.7320	0.6667	0.3350	57	71	26	38	192
	P	0.7473	0.7071	0.7263	0.4540	68	70	29	23	190
	C	0.9063	0.8842	0.8953	0.7907	87	84	11	9	191
	V	0.9500	0.8791	0.9162	0.8333	95	80	11	5	191
9	A	0.7021	0.6771	0.6895	0.3793	66	65	31	28	190
	P	0.7426	0.6882	0.7165	0.4315	75	64	29	26	194
	C	0.9388	0.7935	0.8684	0.7425	92	73	19	6	190
	V	0.8427	0.8614	0.8526	0.7041	75	87	14	14	190
10	A	0.6632	0.7097	0.6862	0.3732	63	66	27	32	188
	P	0.7468	0.6789	0.7074	0.4203	59	74	35	20	188
	C	0.8600	0.7979	0.8299	0.6598	86	75	19	14	194
	V	0.8796	0.8953	0.8866	0.7720	95	77	9	13	194

* A = active training set; P = passive training set; C = calibration set; V = validation set; TN = true negative; TP = true positive; FN = false negative; FP = false positive; All = the total number of compounds in a set.

**Table 3:** A collection of promoters for the increase or decrease of dermal toxicity observed in five processes of the Monte Carlo optimization for split 1.

SMILES Attribute	CWs Probe 1	CWs Probe 2	CWs Probe 3	CWs Probe 4	CWs Probe 5	NA *	NP	NC	d_k_ (Equation (16))
C...C...(...	0.4282	1.1484	0.6594	0.7111	0.8434	89	88	95	0.0003
c...1...c...	0.3818	1.6982	0.0302	0.3192	1.1050	87	81	89	0.0004
C...(...=...	0.3031	0.2886	0.9226	0.1488	0.1215	74	78	74	0.0001
1...c...(...	0.6079	0.2321	0.0176	0.1939	0.4596	50	55	45	0.0006
Cl..........	0.4025	1.4493	1.2160	0.3778	0.4965	44	34	44	0.0009
O...C...C...	0.8311	0.1321	0.3925	0.5969	0.5581	43	37	63	0.0020
c...(...O...	0.8902	0.7246	0.9960	1.0199	0.6417	43	36	35	0.0007
N...C.......	1.6906	2.3710	1.4314	1.4542	0.9820	41	62	47	0.0014
(...Cl..(...	0.5734	0.1407	1.4143	0.4497	0.2051	35	25	36	0.0012
C...(...(...	0.6726	1.1731	1.2948	0.8587	1.4233	33	28	22	0.0014
N...(...C...	1.5790	0.6892	0.4159	1.6733	1.1905	31	35	27	0.0008
c...O.......	0.9687	0.8699	0.9720	1.4256	1.0048	27	20	34	0.0019
=...C...(...	0.9270	0.6279	0.6237	0.7499	1.2820	21	39	23	0.0022
C...#.......	0.9110	0.7571	0.8295	0.8373	0.6587	19	15	13	0.0013
C...C...=...	1.7071	1.8631	0.7322	0.8255	1.1005	19	24	16	0.0014
C...........	−0.2116	−0.5459	−0.4764	−0.4084	−0.1354	183	186	184	0.0000
(...........	−0.1254	−0.7692	−0.3606	−0.2885	−0.3416	164	165	173	0.0002
=...........	−0.6579	−0.1147	−0.3085	−0.2914	−0.2637	111	129	139	0.0008
N...(.......	−0.9757	−0.4264	−0.3162	−0.6883	−0.5884	51	57	52	0.0004
Cl..(.......	−0.3343	−0.2207	−0.8490	−0.5057	−0.1271	36	30	41	0.0011
S...........	−0.8896	−0.6718	−1.0932	−1.1870	−0.0093	28	32	52	0.0023
(...c...(...	−0.7161	−0.3445	−0.7610	−0.8026	−0.9100	18	17	25	0.0014
N...C...(...	−1.4065	−1.3928	−0.4576	−0.8499	−0.6631	18	25	17	0.0013
n...........	−0.5006	−0.4051	−0.5518	−0.2254	−0.2566	18	21	18	0.0005
N...#.......	−1.6835	−1.7643	−1.1689	−1.7475	−1.4276	15	12	10	0.0014
n...c.......	−0.9213	−0.1947	−0.6151	−1.1517	−0.2367	15	16	15	0.0002
c...1...(...	−1.0012	−2.0611	−2.2902	−1.1616	−1.6373	13	21	14	0.0017
=...(...(...	−0.2949	−0.2031	−0.5799	−0.8272	−0.2128	10	17	23	0.0027
S...=.......	−1.0649	−0.1770	−0.5255	−1.5191	−2.6799	10	11	26	0.0036
n...1.......	−1.2993	−0.6785	−0.5283	−0.8285	−0.8261	9	14	9	0.0016

* NA, NP, and NC are the frequencies of SMILES-attributes in the active training, passive training, and calibration sets, respectively; d_k_ is the statistical defect of SMILES-attribute calculated using Equation (16).

**Table 4:** The statistical quality of models for acute dermal toxicity.

Sensitivity	Specificity	Reference
0.74	0.78	[15]
0.71	0.81	[26]
0.89	0.94	[27]
0.88 ± 0.04	0.87 ± 0.03	The average and dispersion on validation sets on models suggested here

## Data Availability

Data are available in the article or its Supplementary Materials.

## Supplementary Materials

The following supporting information can be downloaded at: https://www.mdpi.com/article/10.3390/toxics13040235/s1, Tables S1–S10 contain the statistical characteristics of models observed for 10 splits in the case of optimization with target function TF_1_. The supplementary materials section contains the compositions of all ten sets considered. The active compounds are the same (382), but the inactive ones were selected from the entire set (2234 inactive substances) randomly (also 382 substances each time). For each split, the compounds included in the active training set (A), passive training set (P), calibration set (K), and validation set (V) are indicated.
